# Insights into PBDE Uptake, Body Burden, and Elimination Gained from Australian Age–Concentration Trends Observed Shortly after Peak Exposure

**DOI:** 10.1289/ehp.1408960

**Published:** 2015-03-13

**Authors:** Tenzing Gyalpo, Leisa-Maree Toms, Jochen F. Mueller, Fiona A. Harden, Martin Scheringer, Konrad Hungerbühler

**Affiliations:** 1 Safety and Environmental Technology Group, Swiss Federal Institute of Technology Zurich (ETH Zurich), Zurich, Switzerland; 2 School of Public Health and Social Work and Institute of Health and Biomedical Innovation, Queensland University of Technology, Brisbane, Queensland, Australia; 3 National Research Centre for Environmental Toxicology, The University of Queensland, Brisbane, Queensland, Australia; 4 School of Clinical Sciences and Institute of Health and Biomedical Innovation, Queensland University of Technology, Brisbane, Queensland, Australia; 5 Institute of Sustainable and Environmental Chemistry, Leuphana University Lüneburg, Lüneburg, Lower Saxony, Germany

## Abstract

**Background:**

Population pharmacokinetic models combined with multiple sets of age–concentration biomonitoring data facilitate back-calculation of chemical uptake rates from biomonitoring data.

**Objectives:**

We back-calculated uptake rates of PBDEs for the Australian population from multiple biomonitoring surveys (top-down) and compared them with uptake rates calculated from dietary intake estimates of PBDEs and PBDE concentrations in dust (bottom-up).

**Methods:**

Using three sets of PBDE elimination half-lives, we applied a population pharmacokinetic model to the PBDE biomonitoring data measured between 2002–2003 and 2010–2011 to derive the top-down uptake rates of four key PBDE congeners and six age groups. For the bottom-up approach, we used PBDE concentrations measured around 2005.

**Results:**

Top-down uptake rates of Σ_4_BDE (the sum of BDEs 47, 99, 100, and 153) varied from 7.9 to 19 ng/kg/day for toddlers and from 1.2 to 3.0 ng/kg/day for adults; in most cases, they were—for all age groups—higher than the bottom-up uptake rates. The discrepancy was largest for toddlers with factors up to 7–15 depending on the congener. Despite different elimination half-lives of the four congeners, the age–concentration trends showed no increase in concentration with age and were similar for all congeners.

**Conclusions:**

In the bottom-up approach, PBDE uptake is underestimated; currently known pathways are not sufficient to explain measured PBDE concentrations, especially in young children. Although PBDE exposure of toddlers has declined in the past years, pre- and postnatal exposure to PBDEs has remained almost constant because the mothers’ PBDE body burden has not yet decreased substantially.

**Citation:**

Gyalpo T, Toms LM, Mueller JF, Harden FA, Scheringer M, Hungerbühler K. 2015. Insights into PBDE uptake, body burden, and elimination gained from Australian age–concentration trends observed shortly after peak exposure. Environ Health Perspect 123:978–984; http://dx.doi.org/10.1289/ehp.1408960

## Introduction

At present, there are comprehensive empirical data sets showing the levels of legacy persistent organic pollutants (POPs), such as polychlorinated biphenyls (PCBs) and dichlorodiphenyltrichloroethane (DDT), in human tissue. In combination with time-variant population pharmacokinetic (PK) models, these data have been used to estimate the human intrinsic elimination half-lives of these chemicals and to reproduce cross-sectional age–concentration profiles for the past and future (Quinn and Wania 2012; Ritter et al. 2009, 2011).

For polybrominated diphenyl ethers (PBDEs), the situation differs in several respects. First, most PBDE measurements in humans and in the environment were initiated after 2000 and, therefore, the temporal range of the empirical data is rather short (< 15 years). Second, the timing of these measurements coincides with regulatory efforts to reduce human exposure to PBDEs in different countries and the eventual ban of the commercial mixtures of penta- and octaBDE in the European Union (EU) as well as the voluntary withdrawal from the U.S. market, both in 2004 (EU 2003; U.S. Environmental Protection Agency 2009). Third, nondietary exposure, such as ingestion of residential dust and mouthing behavior, has been found to be as important as or even more important than dietary intake for the total human exposure to PBDEs (Jones-Otazo et al. 2005; Lorber 2008; Stapleton et al. 2012). In contrast, the human exposure to legacy POPs occurs primarily through consumption of fish, meat, and dairy products. However, the contribution of each PBDE exposure pathway has not yet been fully elucidated and, in addition, varies with age and geography (Besis and Samara 2012; Jones-Otazo et al. 2005). Further, in contrast to legacy POPs, no increase in concentration has been observed with increasing age in cross-sectional biomonitoring data; on the contrary, children show higher PBDE concentration than adults (Mueller and Toms 2010). Finally, whereas for legacy POPs a good agreement between measured and modeled concentrations in humans has been observed (Ritter et al. 2009, 2011), this is not the case for PBDEs in the United States (Wong et al. 2013) and in Australia (Toms et al. 2008).

In Australia, the first biomonitoring survey of PBDE levels in the general population that accounted for regional differences, age, and sex was conducted in 2002–2003. Subsequently, four more surveys were conducted in approximately 2-year intervals (Toms et al. 2008, 2009c, 2012, unpublished data). In this period of nearly 10 years, the PBDE concentrations were rather stable in the adult population (age groups 16–30, 31–45, 46–60, > 60 years) from survey to survey [the sum of BDEs 47, 99, 100, and 153 (Σ_4_BDE) was around 10 ng/g lipid in serum samples], whereas in children in the 0–4 years age group, PBDE concentrations declined by two-thirds from 2004–2005 to 2010–2011.

In a first attempt to explain these body concentrations, Toms et al. (2008) compared predicted PBDE body concentrations with the biomonitoring data from the survey of 2004–2005 and discovered a mismatch with the measured levels, the latter being significantly higher than the predicted concentrations. The measured levels could not be explained by the uptake rates calculated by Toms et al. (2008) from PBDE concentrations in contact media and corresponding contact rates. The authors suggested that exposure pathways and/or sources might be missing in the prediction or that the intrinsic elimination half-lives of PBDEs in humans are underestimated.

The intrinsic elimination half-life in the body plays an important role in the balance between intake and body burden. However, until now there have been only three sets of PBDE half-life estimates for the general human population (Geyer et al. 2004; Trudel et al. 2011). In earlier studies, steady-state calculations were performed with either of the two sets of elimination half-lives presented by Geyer et al. (2004) (Table 1) in order to compare predicted with measured PBDE concentration in adults (Abdallah and Harrad 2014; Fromme et al. 2009; Lorber 2008; Toms et al. 2008).

**Table 1:** Intrinsic elimination half-lives (years) estimated for adults with background exposure (general population) as they are used in different scenarios investigated in this study.

Congener	Scenario A^*a*^	Scenario B^*b*^	Scenario C^*c*^
BDE-47	1.4	1.8	3.0
BDE-99	0.8	2.9	5.4
BDE-100	1.8	1.6	2.9
BDE-153	7.4	6.5	11.7
^***a***^Trudel et al. (2011): based on steady-state assumption, uptake from eight exposure pathways, human population: North America and Europe. ^***b***^Geyer et al. (2004): based on steady-state assumption, dietary uptake only, human population: Sweden. ^***c***^Geyer et al. (2004): extrapolation from experimental rat studies.			

In the present study, we systematically investigated the gap between PBDE uptake rates derived from PBDE levels measured in the Australian population and PBDE uptake rates derived from exposure to PBDEs in food, air, and dust. To that end, we employed a time-variant population PK model using the data sets of five biomonitoring surveys performed between 2002–2003 and 2010–2011. Our goals were *a*) to back-calculate total daily uptake of four key PBDE congeners (BDEs 47, 99, 100, and 153) for different age groups (0–3 months, 3–12 months, 1–6 years, 6–12 years, 12–20 years, and > 20 years) of the Australian population based on the biomonitoring surveys using different elimination half-lives (“top-down” approach); *b*) to calculate “bottom-up” uptake rates from diet, dust ingestion, dermal absorption, and inhalation, and compare these uptake rates to those derived from the top-down approach; and *c*) to provide guidance on the interpretation of cross-sectional biomonitoring data of lipophilic POPs regarding accumulation with age and derivation of the chemical’s elimination half-life.

## Methods

*Biomonitoring data*. We used congener-specific cross-sectional data of four key PBDE congeners, BDE-47, BDE-99, BDE-100, and BDE-153, sampled in the following biomonitoring surveys in Australia: 2002–2003 [60 pooled samples (p.s.)], 2004–2005 (12 p.s.), 2006–2007 (81 p.s.), 2008–2009 (12 p.s.), and 2010–2011 (12 p.s.). Blood serum samples of > 15,000 residents were collected and pooled for the analysis of individual PBDE congeners (in nanograms per gram of lipid). The pools were stratified by age and sex; each pool represents the average concentration of 30 serum samples (survey in 2006–2007) or up to 100 serum samples (all other surveys). Age groups common to all surveys were 16–30, 31–45, 46–60, and > 60 years. In addition, age groups of 0–4 and 5–15 years were available for the surveys in 2004–2005, 2008–2009, and 2010–2011; in 2002–2003, the youngest age group was < 16 years. In 2006–2007, the age groups covered 6-month periods from newborn to 4 years of age, and were followed by 3-year periods for ages 4–15 years. A detailed description of the analytical method is provided elsewhere (Toms et al. 2008, 2009c, 2012, unpublished data).

For the present analysis, we used the concentrations of the following age groups for the back-calculation of PBDE exposure 0–4, 5–15, < 16, 16–30, 31–45, 46–60, and > 60 years. For the sake of consistency and equal weighting, the eight age groups of 6-month periods and the four age groups of 3-year periods in the survey of 2006–2007 were randomly combined to represent the age groups of 0–4 and 5–15 years, respectively.

**Figure 1:**
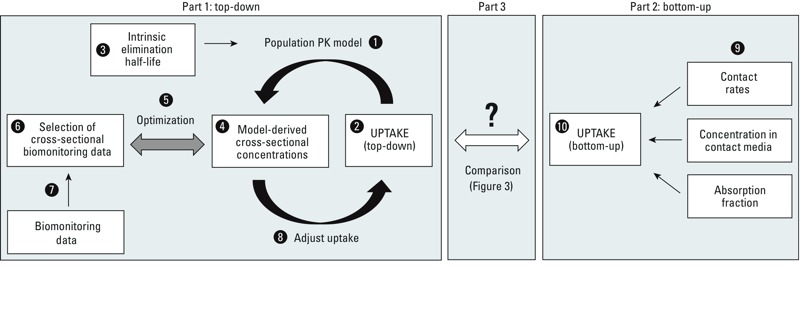
Overview of the approach employed in this work.

*Part 1. Top-down approach for uptake*. The time-variant population PK model originally presented by Ritter et al. (2011) was applied (step 1 in Figure 1). All model equations are provided in the Supplemental Material (Equations S1–S4). Briefly, single individuals are represented as a single well-mixed lipid compartment that receives PBDEs via uptake and loses PBDEs via elimination (excretion and/or metabolism). The size of the lipid compartment varies as a function of age and reflects age-dependent changes in body weight and lipid fraction (Australian Bureau of Statistics 1998; International Commission on Radiological Protection 2002; World Health Organization 2006). The PK model calculates longitudinal concentrations of chemicals as a function of age for representative females and males born in 1-year intervals from 1921 until 2020. Uptake rates (step 2) are age and time dependent and represent the internal dose, (i.e., the amount of chemical that passes absorption barriers such as skin, lung tissue, and gastrointestinal tract wall), whereas the chemicals’ intrinsic elimination half-lives are age-independent (step 3). These two parameters define the longitudinal PBDE concentration profile in each individual.

For comparison with empirical data, cross-sectional concentrations (step 4 in Figure 1) were extracted from the PK model representing concentrations of individuals of different ages at the same calendar time. These model-derived cross-sectional trends were then compared (step 5) with a subset of the biomonitoring data (step 6), which is a random sample (step 7) from the full set of biomonitoring data. As long as the agreement could be improved, the uptake rate was adjusted (step 8) and the PK model was re-run.

Time-dependent uptake. Few data are available for the parameterization of a time-variant uptake of PBDEs in Australia. The time-variant adult reference uptake was described by an exponential increase during the phase of production and use followed by an exponential decrease after import stop or ban of the chemicals. We assumed that the transition happened in 2001 (prior to the actual ban in 2005) because the importation of penta- and octaBDE mixtures in Australia dropped significantly after 1998–1999, from 72 to 10–30 metric tons/year for pentaBDE and from 47 to < 10 metric tons/year for octaBDE in 2003–2004 (National Industrial Chemicals Notification and Assessment Scheme 2005). The exposure half-life (i.e., halving time) after 2001 was derived from the declining trend in PBDE concentrations in dust samples in Australia; in quantifying this trend, we included only studies with ≥ 10 investigated homes (Sjödin et al. 2008; Stasinska et al. 2013; Toms et al. 2009b). No trend data of other exposure-related parameters were available for Australia. Between 2004 and 2010, the concentrations in dust declined with half-lives varying between 5.9 and 9.0 years for the four PBDE congeners. On the basis of these data, we used the average of 7 years to represent the half-life of declining PBDE exposure for all congeners. Because no data on increases in PBDE exposure prior to 2001 in Australia are available, we applied the approach by Wong et al. (2013) and mirrored the trend in decline; that is, we used an exposure doubling time of 7 years for all congeners.

Age-dependent uptake. The initial PBDE concentration of newborns was set to be equal to the maternal concentration. Newborns were assumed to be exclusively breastfed for 3 months (Australian Institute of Health and Welfare 2011), and transfer of chemical from the mother to the newborn was modeled as shown by Verner et al. (2013). Because all PBDE concentrations are lipid-normalized, the mother’s concentration of PBDEs was used as the PBDE concentration in breast milk. For the age groups older than newborns, uptake rates were obtained by multiplying the adult reference uptake by a proportionality factor derived from PBDE intake reported by Lorber (2008) (see Supplemental Material, Figure S1 and Table S1).

Optimization process. In the optimization, the only adjustable parameter was the adult reference uptake rate in 2001. It was varied in a least-squares optimization until the difference between modeled PBDE levels and measured levels was minimal (see Supplemental Material, Equation S4). The modeled PBDE concentrations are the average concentrations of groups of modeled individuals that include the same number of individuals and the same average age as the pools from which the measured PBDE concentrations were obtained in the biomonitoring surveys.

**Figure 2:**
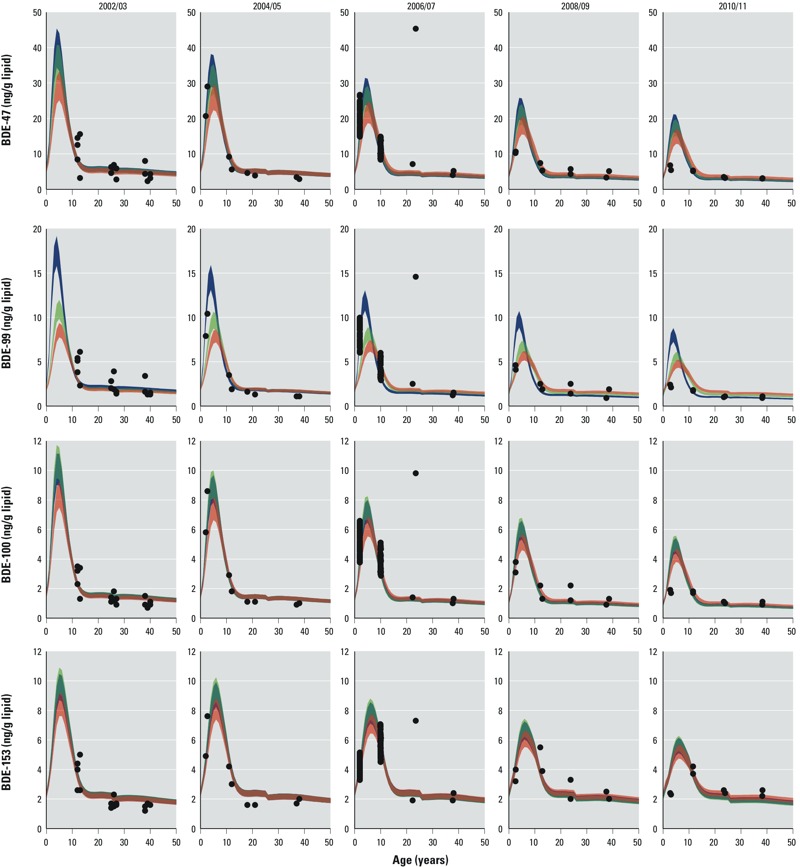
Modeled age–concentration profiles (blue: scenario A; green: scenario B; red: scenario C) fitted to the biomonitoring data (dots) of BDE-47, BDE-99, BDE-100, and BDE-153 in the female population. To increase the visibility of the data for the younger age groups, the *x*-axis ends at 50 years and the two oldest age groups are not shown. The concentrations in adults > 50 years of age equal the concentrations of adults < 50 years of age and therefore do not contribute additional information.

**Figure 3:**
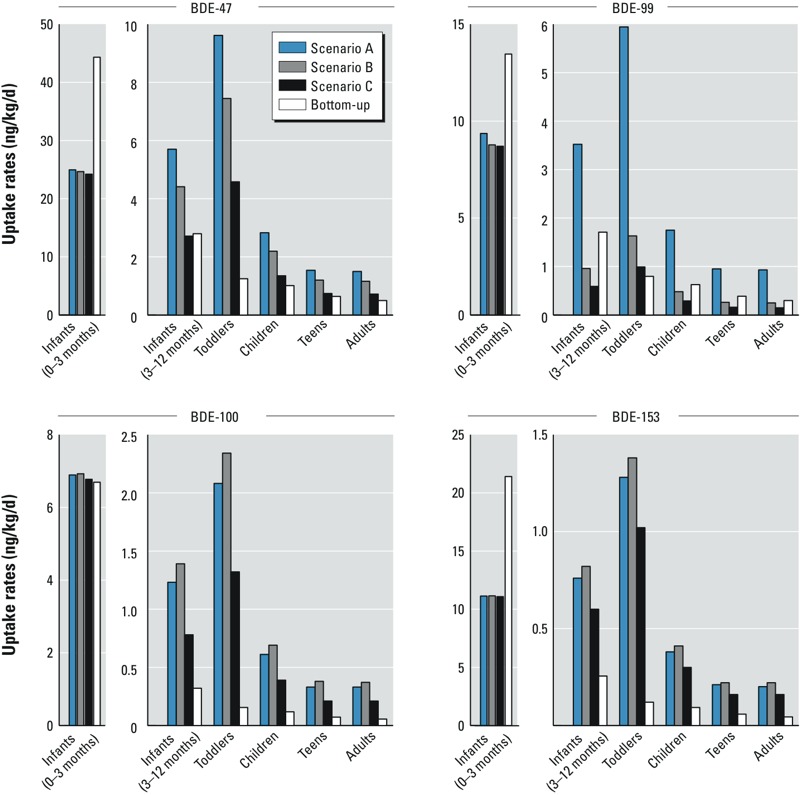
PBDE uptake rates of different age groups in 2005 derived from both the top-down approach with different elimination half-lives and the bottom-up approach. Note the different scale on the *y*-axis for infants 0–3 months of age.

Bootstrapping. To preserve the empirical variability in the biomonitoring data, we ran the optimization process 100 times with 100 different biomonitoring data subsets for each PBDE congener and each elimination half-life (scenarios A, B, and C; Table 1). Each subset was bootstrapped (step 7 in Figure 1) from the full set of biomonitoring data; we randomly selected 1 pool per age group and survey (throughout the five surveys, the number of pools per age group varied from 2 to 9). Each subset consisted of 29 pools (five age groups from survey 2002–2003; six age groups from the other surveys). The variability in body concentrations originating from the 100 simulations is shown as shaded areas for the female population in Figure 2. The 100 simulations resulted in 100 optimized uptake rates; the average uptake rates for each age group for the year 2005 are shown in Figure 3. We refer to the uptake rates from the model fit as “top-down” uptake rates.

*Part 2. Bottom-up approach for uptake*. For comparison with the top-down uptake rates (Part 3 in Figure 1), we calculated uptake rates using the bottom-up approach. Estimates of PBDE dietary intake and PBDE concentrations in office dust were available only for 2005 [Food Standards Australia New Zealand (FSANZ) 2007; Toms et al. 2009a]. In combination with generic contact rates and absorption factors (step 9 in Figure 1), we estimated total uptake rates (step 10) from diet (including breast milk), dust ingestion (home and office), dermal uptake from dust, and indoor air inhalation (home and office) (see Supplemental Material, Tables S2 and S3).

Sensitivity and uncertainty analysis. We examined the effect of four model parameters that potentially influence the optimized uptake rates. First, the variability in the intrinsic elimination half-lives was accounted for by running the PK model for each congener with three different elimination half-lives (scenarios A, B, and C; Table 1). Further, we tested *a*) the influence of the year of peak exposure (by moving it from 2001 to 1998 and 2004), *b*) the exposure doubling and halving time (by changing it from 7 to 4.5 years), and *c*) the proportionality factor for age-dependent uptake rates by using a different PBDE exposure study for its derivation (see Supplemental Material, Figure S1 and Table S1).

## Results

Figure 2 presents the fitted PBDE body burdens of the female population for scenarios A, B, and C and for each PBDE congener and sampling year. The shaded areas represent the variability of the 100 bootstrapping runs. Results for the male population are shown in Supplemental Material, Figure S2.

For all congeners and all elimination half-lives, there is the same course of concentration versus age within each survey: The modeled cross-sectional concentration in the population increases from birth until the age of 4–6 years for BDE-47, -99, and -100, and until 5–8 years for BDE-153, and thereafter decreases and levels off during adulthood. Further, from year to year the peak concentration in children decreases because, in contrast to adults, children have no exposure from earlier years and thus their body burden is directly determined by the current uptake rate. This means that decreasing exposure following the peak in 2001 has a greater effect on PBDE concentrations in children than in adults.

Figure 3 presents the average fitted top-down uptake rates derived from the different model scenarios A, B, and C, as well as the uptake rates from the bottom-up approach for the different age groups of the Australian population for the year 2005. The exact values are presented in Supplemental Material, Table S4.

*Top-down uptake*. The highest fitted uptake rates for all age groups are present in the scenarios with the shortest elimination half-lives, that is, BDE-47 and BDE-99 in scenario A, and BDE-100 and BDE-153 in scenario B (Figure 3). The fitted uptake rates decrease with increasing elimination half-lives. For BDE-47, uptake rates in scenarios B and C are 75% and 50%, respectively, of those in scenario A for all age groups. For BDE-99, they are 30% and 20%. For BDE-100, scenario B yields 115% and scenario C 65% of the uptake rate in scenario A. The same trend is seen for BDE-153, where uptake rates in scenario B and scenario C are 110% and 80%, respectively, of those in scenario A. However, there is virtually no difference in the uptake rates of breastfed infants among all scenarios (Figure 3, Table 2), which is due to the fact that the modeled PBDE concentration in the breast milk of the 25-year-old mothers is very similar in all three scenarios (Figure 2).

**Table 2:** ∑_4_BDE uptake rates (ng/kg/day).

Age group	Scenario A	Scenario B	Scenario C	Bottom-up
Infants (0–3 months)	52	51	51	86
Infants (3–12 months)	11	7.6	4.7	5.1
Toddlers	19	13	7.9	2.3
Children	5.6	3.8	2.3	1.8
Teens	3.0	2.1	1.3	1.2
Adults	3.0	2.0	1.2	0.89

*Age groups*. Due to exclusive breastfeeding, infants younger than 3 months of age have the highest PBDE uptake rate in all scenarios (Figure 3). For infants older than 3 months, the uptake rate decreases rapidly by factors of 3–15, 5–14, and 9–18, respectively, depending on the PBDE congener in scenarios A, B, and C, with BDE-153 showing the largest differences (see Supplemental Material, Table S4). With increasing age, the uptake rates continue to decline to levels of 3.0, 2.0, and 1.2 ng/kg/day of Σ_4_BDE for adults in scenarios A, B, and C, respectively (Table 2).

*Bottom-up uptake*. A similar age trend is visible in the uptake rates from the bottom-up approach (Figure 3). Breastfed infants have the highest uptake rates (Table 2); for the different PBDE congeners, they are 8–84 times higher than the uptake rate of infants above 3 months of age (see Supplemental Material, Table S4). Again, the difference is highest for BDE-153. Σ_4_BDE uptake rates for toddlers, children, and teens range from 1.2 to 2.3 ng/kg/day, with toddlers having the highest exposure (Table 2). Adults have the lowest Σ_4_BDE uptake rate of 0.89 ng/kg/day.

For the top-down approach, it is not possible to break down PBDE uptake rates in terms of different exposure pathways, but this can be done for the bottom-up approach. For all age groups, dietary uptake is by far the most important exposure pathway, representing 85–95% of total uptake for all age groups. Dust ingestion is responsible for the remaining uptake; inhalation and dermal uptake are negligible (data not shown).

*Bottom-up versus top-down approach*. In general, the top-down uptake rates are higher than the bottom-up uptake rates (Figure 3, Table 2). For all age groups except breastfed infants, the top-down uptake rates are higher than the bottom-up uptake rates by factors of 2–8, 2–7, 2–15, and 2–12 for BDE-47 (scenarios A and B), BDE-99 (scenario A only), BDE-100, and BDE-153, respectively, depending on the age group and scenario (see Supplemental Material, Table S4). The largest differences, on the order of a factor of 10, are found for toddlers. In terms of PBDE congeners, the differences in uptake rates are higher for BDE-100 and BDE-153 than for BDE-47 and BDE-99. Even with the longest set of elimination half-lives (scenario C), the top-down uptake rates of BDE-100 and BDE-153 for adults are still four times higher than their corresponding bottom-up uptake rates.

It is important to note that in cases where top-down and bottom-up uptake rates are similar (i.e., BDE-99 in scenarios B and C, and BDE-47 in scenario C), the modeled concentrations are clearly lower than the measured concentrations for children (Figure 2; see also Supplemental Material, Figure S2). This suggests that the top-down uptake rates are too low in these cases and that the actual uptake rates are higher than indicated by both top-down and bottom-up uptake rates.

## Discussion

Ideally, there would be agreement between modeled and measured cross-sectional concentrations for all biomonitoring surveys, as well as agreement between top-down and bottom-up uptake rates. Because this is not the case for the investigated PBDE congeners and any of the scenarios, we conclude that either some exposure sources and/or pathways are missing or underestimated in the bottom-up approach or that the current intrinsic PBDE elimination half-lives are underestimated, or a combination of both. This is in agreement with the findings of Toms et al. (2008).

*Top-down approach: sensitivity and uncertainty analysis*. Overall, the elimination half-lives of the four key PBDE congeners had the largest effect on the uptake rates for 2005 (Figure 3). In contrast, the choice of the exposure doubling and halving time had little effect on the uptake rates for 2005 (a deviation of only ± 3%) (data not shown). Next, moving the year of maximum uptake from 2001 to 1998 resulted in a reduction in uptake rates of 7–15% depending on the scenario, whereas a shift from 2001 to 2004 resulted in an increase of 18–29% (data not shown). Finally, by using a different PBDE exposure study (Trudel et al. 2011) for the derivation of the proportionality factor, the uptake rates of toddlers were most affected among the different age groups. Their uptake rates decreased by 37% because the average proportionality factor was reduced from 6.4 to 3.8 for this age group; at the same time, the adult uptake rates increased by 7% (data not shown). Altogether, we altered influential model parameters within reasonable ranges. The highest effects on the top-down uptake rates across all model scenarios were a factor of 1.4–6, caused by the changes in elimination half-lives (see Supplemental Material, Table S4, for scenarios A–C), and a factor of 0.6 (decrease by 37%) to 1.3 (increase by 30%) caused by changes in other parameters (data not shown). In contrast, the difference between top-down and bottom-up uptake rates are up to a factor of 15 (see Supplemental Material, Table S4).

*Bottom-up approach: uncertainties*. In our bottom-up approach, the predominant exposure pathway is diet, which in all age groups contributes 85–95% of the Σ_4_BDE uptake (calculated as described in Part 2 in “Methods”). According to FSANZ (2007), bread and boiled eggs are the most important food items, together contributing approximately 30% to the total dietary intake, independent of age group. Fish, in contrast, contributes only 1–2% (FSANZ 2007). Also in many European and Asian countries, dietary intake is the main PBDE exposure pathway for the general population, but the largest contribution, up to 67% of the total dietary intake, stems from fish and shellfish (Domingo 2012; Na et al. 2013). Thus, this particular food category might be substantially underestimated in Australia. Strong evidence that this might be the case is given by the similar fish and seafood consumption rates in Australia and Western Europe (National Oceanic and Atmospheric Administration 2012) and even higher PBDE contamination in fish consumed in Australia than in Europe (Domingo 2012).

The contribution of PBDE exposure from dust to the total uptake for the Australian population is low (16% for toddlers, 6% for adults; calculated as described in Part 2 in “Methods”). A possible explanation of the discrepancy between top-down and bottom-up uptake rates could be a large underestimation of the PBDE concentrations in the dust samples from Australia. However, the dust samples (*n* = 5–30) measured between 2004 and 2012 in homes, offices, and schools (Sjödin et al. 2008; Stasinska et al. 2013; Toms et al. 2009a, 2009b, 2015) show similar mean and median concentrations of the key congeners and are much closer to the PBDE concentrations found in Europe than to those in North America (Whitehead et al. 2011). It is unlikely that all of these measurements systematically underestimated the actual PBDE levels in dust in Australia.

Had we used concentrations of 2,000, 1,000, 500, and 150 ng/g dust for BDEs 47, 99, 100, and 153 in dust samples, respectively, which are in the range of the U.S. data, the total Σ_4_BDE uptake rates would increase from 2.3 to 6.8 ng/kg/day for toddlers and from 0.89 to 1.5 ng/kg/day for adults. This approximately 10-fold increase in hypothetical average PBDE concentrations in dust samples would produce good agreement between top-down and bottom-up uptake rates for all age groups except for toddlers. However, as noted above, such elevated average PBDE concentrations in dust samples are not supported by the PBDE measurements in dust from Australia.

Our bottom-up uptake rates are more uncertain than the top-down uptake rates because the underlying sample sizes are small. Dietary intake is, according to our analysis, the dominant exposure pathway, but dietary intake estimates were determined only once (in 2005) (FSANZ 2007). In addition, dust ingestion rates are very uncertain parameters and vary substantially between exposure studies; specifically, values used for toddlers vary from 50 to 200 mg/day (here 60 mg/day) and for adults from 4.16 to 100 mg/day (here 30 mg/day) (Jones-Otazo et al. 2005; Toms et al. 2009a).

*Why the PBDE concentrations do not increase with age*. The present situation with PBDEs is similar to the situation with PCBs shortly after 1970, for which Quinn and Wania (2012) modeled the cross-sectional age–concentration trends. For the transition period after the peak exposure in 1974, the course of the modeled cross-sectional data of PCB-153 (elimination half-life, 15 years) looks like the current course of the PBDE concentrations in the Australian population, as shown in Figure 2. This is the case although the time trends of the PBDEs shown in Figure 2 are based on elimination half-lives considerably shorter than 15 years. That is, cross-sectional biomonitoring data of lipophilic chemicals (independently of a chemical’s elimination half-life) exhibit age–concentration profiles as presented in Figure 2 if they are sampled during the transition period, that is, within 10 years after peak exposure (Quinn and Wania 2012).

It is not until at least 20 years after the year of peak exposure that the age–concentration profile of chemicals with long elimination half-lives starts to differentiate from that of rapidly eliminated chemicals (Quinn and Wania 2012; Ritter et al. 2011). In this later stage, the concentrations of chemicals with long elimination half-lives increase with increasing age, which is not the case for chemicals with rapid elimination. Thus, now we observe chemical concentrations increasing with age in cross-sectional biomonitoring data for legacy POPs such as most PCBs, dioxins, and dichlorodiphenyldichloroethylene (DDE) (Mueller and Toms 2010), but not (yet) for PBDEs (Garí and Grimalt 2013; Sjödin et al. 2013; Thomsen et al. 2002; Toms et al. 2012).

*Why we cannot fit the elimination half-lives of PBDEs*. It is important to note that the currently available PBDE biomonitoring data from 2002–2003 to 2010–2011 cannot be used to accurately estimate the PBDE elimination half-lives because these data were collected in the transition phase around the time of maximum exposure. As stated above, in this phase, age–concentration profiles show the same trend independently of the chemical’s elimination half-life.

This is different from the analysis performed by Ritter et al. (2011), who used PCB biomonitoring data to estimate PCB elimination half-lives in humans by fitting a population PK model to the biomonitoring data. In their case, the data were from 1990 and 2003, but the ban of PCBs had taken place already in the 1970s. Both PCB data sets were sampled in the post-ban phase two and three decades after the ban. Under these conditions, the data were sufficient to constrain congener-specific elimination half-lives for different PCBs because, in the long term, the age–concentration profile shows an increase in concentrations with increasing age if the chemical’s elimination half-life is longer than the exposure half-life. If not, the age–concentration profiles are the same as they are observed today for PBDEs; that is, there is no increase with age, as it was found for PCB-52 (Ritter et al. 2011).

Therefore, derivation of the elimination half-life from the current PBDE biomonitoring data would result in an underestimation of the elimination half-life of those chemicals that truly have long elimination half-lives because the PK model is not constrained by long-term biomonitoring data, as was the case for PCBs (Ritter et al. 2011).

## Conclusions

We have provided new evidence for the inconsistency between uptake rates derived from biomonitoring data and uptake rates calculated from dietary intake of PBDEs and PBDE concentrations in dust. Especially the contribution from fish and shellfish might currently be highly underestimated in Australia. Therefore, long-term continuation of biomonitoring surveys of identical design and complemented with measurements of PBDE concentrations in contact media are vital for identifying the cause of the mismatch between modeled and measured PBDE concentrations and also as a basis of potential measures for risk reduction and risk management.

Beyond the case of PBDEs, insights gained from this study suggest that cross-sectional biomonitoring data of emerging lipophilic chemicals, such as alternative brominated flame retardants [e.g., bis-(tribromophenoxy)ethane (BTBPE) or decabromodiphenyl ethane (DBDPE)] will show the same age–concentration profile as observed in the current biomonitoring data of PBDEs, that is, no increase in concentration with increasing age (Figure 2).

Further, it is important to note that as a result of the declining exposure, the PBDE body burden of toddlers and children has declined during the past 10 years, whereas the PBDE exposure of fetuses and breast-fed infants (the most sensitive groups) has remained rather constant. This is because the PBDE body burden of the mothers has not reacted as fast as that of young children to the decreasing exposure (Figure 2).

## Supplemental Material

(933 KB) PDF
