# Supplementary material for: Insights into PBDE Uptake, Body Burden, and Elimination Gained from Australian Age–Concentration Trends Observed Shortly after Peak Exposure
Source: Environ Health Perspect. 2015 Mar 13;123(10):978–84. doi: 10.1289/ehp.1408960 (PMC4590757; doi:10.1289/ehp.1408960)
Supplement: (933 KB) PDF [file ehp.1408960.s001.acco.pdf]

Environ Health Perspect
doi: 10.1289/ehp.1408960

Note to Readers: ***EHP*** **strives to ensure that all journal content is accessible to all readers. However, some figures and Supplemental Material published in *EHP* articles may not conform to 508 standards due to the complexity of the information being presented. If you need assistance accessing journal content, please contact ehp508@niehs.nih.gov. Our staff will work with you to assess and meet your accessibility needs within 3 working days.**

Insights into PBDE Uptake, Body Burden, and Elimination Gained from Australian Age–Concentration Trends Observed Shortly after Peak Exposure

Tenzing Gyalpo, Leisa-Maree Toms, Jochen F. Mueller, Fiona A. Harden, Martin Scheringer, and Konrad Hungerbühler

Table of Contents

1. Parameterization of the time-variant population pharmacokinetic (PK) model

Transfer of chemical via breast milk

Proportionality factor

Table S1. Derivation of proportionality factors.

Figure S1. Interpolated proportionality factors $P(t_{age})$ (lines) fitted to empirical proportionality factors (diamonds). A: Lorber (2008); B: Trudel et al. (2011).

Least-squares optimization

2. Modeled and measured cross-sectional age-concentration profile for the male population

Figure S2. Modeled age-concentration profiles (blue: scenario A; green: scenario B; red: scenario C) fitted to the biomonitoring data (dots) from the male population.

3. Input data for the PBDE bottom-up approach

Table S2. Congener-specific parameters.

Table S3. Age-dependent parameters.

4. Congener-specific uptake rates from the model simulations

Table S4. Congener-specific uptake rates (ng kg^{-1} d^{-1}).

References

1. Parameterization of the time-variant population pharmacokinetic (PK) model

Equation 1 defines the time course of the chemical concentration in a representative individual born at time t_{birth}:

$$\frac{dC(t_{\mathrm{age}})}{dt} = \frac{U_{\mathrm{ref}}(t) \cdot M_{\mathrm{bw}}(t_{\mathrm{age}}) \cdot P(t_{\mathrm{age}}) \cdot F}{M_{\mathrm{lip}}(t_{\mathrm{age}})} - \left(k_{\mathrm{elim}} + \frac{1}{M_{\mathrm{lip}}(t_{\mathrm{age}})} \cdot \frac{dM_{\mathrm{lip}}(t_{\mathrm{age}})}{dt}\right) \cdot C(t_{\mathrm{age}}) \qquad [1]$$

where t_{age} (years) is the age of the individual; $C(t_{\mathrm{age}})$ (ng g_{lip}^{-1}) is the lipid-normalized concentration of chemical in the body; $U_{\mathrm{ref}}(t)$ (ng kg^{-1} d^{-1}) is the reference daily uptake of the chemical for an adult and depends on the year of sampling, t; $M_{\mathrm{bw}}(t_{\mathrm{age}})$ (kg) and $M_{\mathrm{lip}}(t_{\mathrm{age}})$ (kg_{lip}) are the body weight and the body lipid weight as a function of age, respectively; $P(t_{\mathrm{age}})$ (dimensionless) is a proportionality factor adapting $U_{\mathrm{ref}}(t)$ to younger ages; F (kg_{lip}^{-1} g_{lip}) is a unit conversion factor; k_{elim} (d^{-1}) is the first-order rate constant describing intrinsic elimination. Importantly, U_{ref} represents the **absorbed** amount of chemicals (= uptake) from all sources and pathways (excluding breast milk) that contribute to the PBDE concentration in the body.

The model was programmed in Matlab R2013a and solved with a 3-day resolution.

Transfer of chemical via breast milk

The daily human milk consumption rate, $r_{\mathrm{bm}}(t_{\mathrm{age}})$ (g d^{-1}) and the lipid fraction of the human milk, $f_{\mathrm{lip,bm}}(t_{\mathrm{age}})$ (dimensionless) are described dependent on the age of the infant (t_{age}) (years) and his/her body weight (M_{bw}) (kg) according to Verner et al. (2013) (Equations 2 and 3):

$$r_{\mathrm{bm}}(t_{\mathrm{age}}) = (-0.0024 \cdot t_{\mathrm{age}} + 0.0063) \cdot M_{\mathrm{bw}}(t_{\mathrm{age}}) \cdot 24 \cdot 1000 \qquad [2]$$

$$f_{\mathrm{lip,bm}}(t_{\mathrm{age}}) = 0.0034 \cdot \ln(t_{\mathrm{age}}) + 0.0414 \qquad [3]$$

Proportionality factor

We derived the proportionality factor, $P(t_{age})$ in Equation 1, by dividing the uptake rates of younger age groups by the uptake rate of adults (Table S1). The empirical proportionality factors show steps, because they represent whole age groups, i.e. 1–6, 6–12, 12–20, and >20 years (Figure S1, black and blue diamonds). Since no exposure estimate is given for infants < 1 years in Lorber (2008), we assumed it to be 50% of that of the group of 1–6 years. We used a Weibull function to interpolate the proportionality factors for uptakes of the different age groups (black and blue lines). We used data from Lorber (2008) as base case (panel A in Figure S1). As an alternative, we used the median uptake rates for the US population from Trudel et al. (2011) (panel B in Figure S1).

Table S1. Derivation of proportionality factors.

Age group	Lorber (2008) ∑PBDE intake (ng kg^{-1} d^{-1})	Factor (unitless)	Trudel et al. (2011)[a] ∑PBDE intake (ng kg^{-1} d^{-1})	Factor (unitless)
infants	49.3/2 = 24.7	3.2	5.5*0.7 = 3.85	3.5
toddlers	49.3	6.4	4.4	4
children	14.4	1.87	2.2	2
teenager	9.1	1.18	1.25	1.14
adult	7.7	1	1.1	1

[a]Table S7 of Trudel et al. (2011).

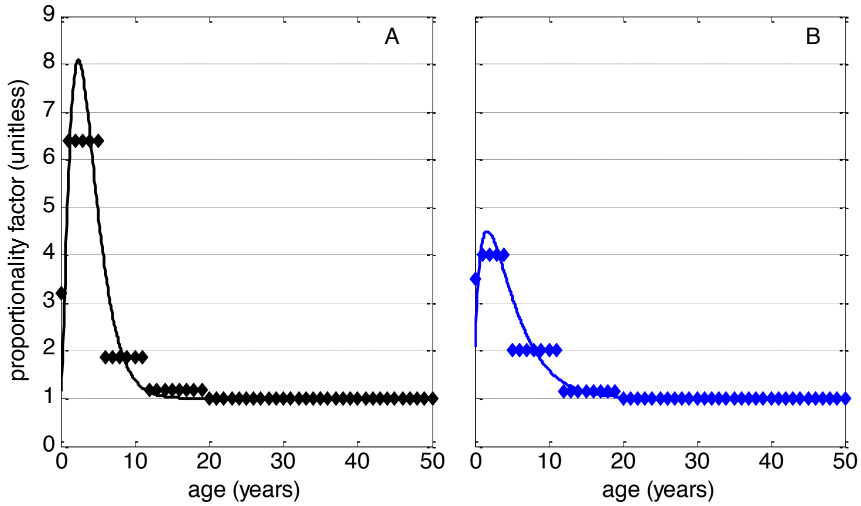

Figure S1. Interpolated proportionality factors $P(t_{age})$ (lines) fitted to empirical proportionality factors (diamonds). A: Lorber (2008); B: Trudel et al. (2011).

Least-squares optimization

For each optimization, we used 29 empirical data points (see main text). By minimizing the sum of squared residuals weighted (SSRW), we maximized R^2 (Equation 4):

$$R^2 = 1 - SSRW = 1 - \frac{\sum_{i=1}^{n}(y_i - f_i)^2}{\sum_{i=1}^{n}(y_i - y)^2} \qquad [4]$$

where n is the number of empirical data points, here $n = 29$, y_i is the empirical data point i, f_i is the equivalent modeled value, and y is the empirical sample mean.

2. Modeled and measured cross-sectional age-concentration profile for the male population

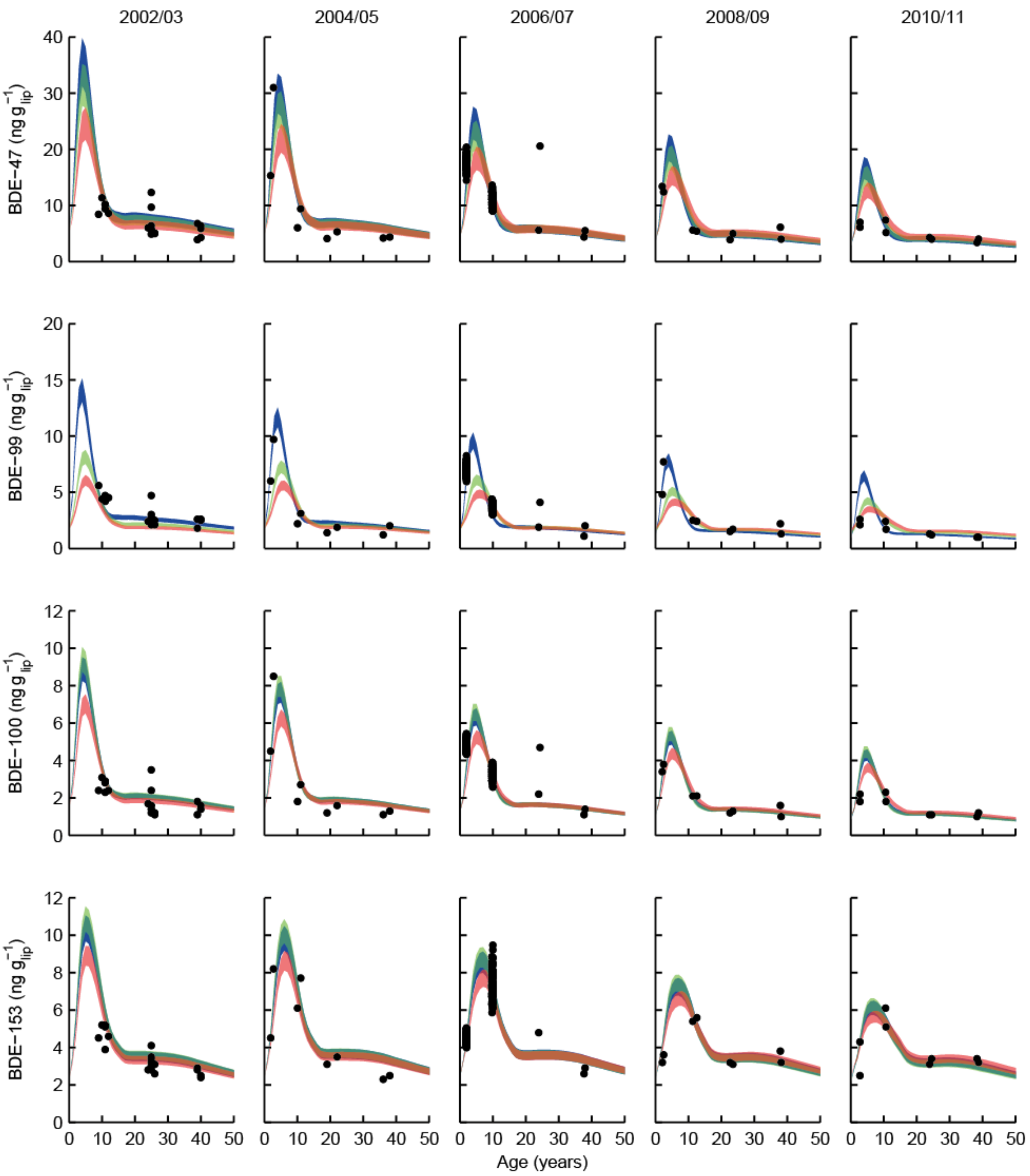

Figure S2. Modeled age-concentration profiles (blue: scenario A; green: scenario B; red: scenario C) fitted to the biomonitoring data (dots) from the male population.

3. Input data for the PBDE bottom-up approach

The lipid fraction of breast milk was set to 3.3% (Toms et al. 2012). The transfer fraction from dust to skin was set to 13% (Trudel et al. 2011); the dermal absorption fraction was set to 3% (Roper et al. 2006).

Table S2. Congener-specific parameters.

	Unit	BDE-47	BDE-99	BDE-100	BDE-153	Reference
concentration in breast milk[a]	ng g_{lip}^{-1}	9.3	2.8	1.4	4.5	Toms et al. (2012)
concentration in dust at home (mean)	ng g^{-1}	69	135	27	18	weighted average of the mean values from Toms et al. (2009a,b)
concentration in dust in offices (mean)	ng d^{-1}	107	153	30	24	Toms et al. (2009a)
concentration in air at home (mean)[a]	ng m^{-3}	0.0286	0.0094	0.0024	0.0023	Toms et al. (2009a)
concentration in air in the office (mean)[a]	ng m^{-3}	0.1298	0.0100	0.0025	0.0010	Toms et al. (2009a)
absorption fraction from breast milk, diet, inhalation	[–]	0.99	0.99	0.99	0.99	Moser & McLachlan (2001)
absorption fraction from dust	[–]	0.58	0.41	0.53	0.48	Abdallah et al. (2012)

[a]Sampled in 2003/04. [b]Assumption: if concentration <LOD, LOD/2 was used.

Table S3. Age-dependent parameters.

	Sex/congener	Infants 0–3 months	infants 3–12 months	Toddlers 1–6 years	Children 6–12 years	Teenagers 12–20 years	Adults >20 years	Reference
body weight, [kg]	males	5	8	17	31	65	82	WHO (2006), ABS (1998)
	females	5	8	17	32	58	68	
fraction of time spent home, [–]		0.77	0.77	0.68	0.62	0.60	0.66	U.S. EPA (2011)
fraction of time spent in the office, [–]		0	0	0	0	0	0.15	Horton et al. (2006)
daily intake of breast milk, [g d^{-1}]		727	0	0	0	0	0	WHO (2002), average of the first 3 months
dust ingestion rate, [g d^{-1}]		0.015[a]	0.03	0.06	0.06	0.06	0.03	U.S. EPA (2011)
dust adhered to skin, [g cm^{-2}]		3.40E-05	3.40E-05	3.40E-05	3.40E-05	8.90E-06	8.90E-06	Trudel et al. (2011), SI
body surface area, [cm^2 d^{-1}]	males	3233	4367	6800	11500	17550	20700	U.S. EPA (2011)
	females	3000	4200	6660	11250	16200	18123	
fraction of free surface area[b], [–]	males	0.22	0.22	0.25	0.27	0.27	0.29	U.S. EPA (2011)
	females	0.22	0.22	0.25	0.27	0.27	0.27	
inhalation rate, [m^3 d^{-1}]	males	3.38	3.94	7.10	10.59	17.23	15.27	U.S. EPA (2011)
	females	3.26	3.73	6.60	9.84	13.28	11.79	
daily intake via diet, [ng d^{-1}] males	BDE-47	0	21.5	20.2	32.1	45.6	43.6	FSANZ (2007)
	BDE-99	0	12.4	11.6	18.5	26.2	25.1	(lower-bound estimates)
	BDE-100	0	2.2	2.1	3.3	4.7	4.5	
	BDE-153	0	1.8	1.7	2.7	3.9	3.7	
daily intake via diet, [ng d^{-1}] females	BDE-47	0	21.5	19.3	27.9	30.8	28.9	FSANZ (2007)
	BDE-99	0	12.4	11.1	16.1	17.7	16.6	(lower-bound estimates)
	BDE-100	0	2.2	2.0	2.9	3.2	3.0	
	BDE-153	0	1.8	1.6	2.4	2.6	2.5	

[a]Assumption; value of <3 months equivalent to 50% of value of 3–12 months. [b]Assumption; free surface area = 50% of arms plus 50% of legs plus hands.

4. Congener-specific uptake rates from the model simulations

Table S4. Congener-specific uptake rates (ng kg^{-1} d^{-1}).

	Age groups	BDE-47	BDE-99	BDE-100	BDE-153
scenario A	infants (0–3 mo)	25	9.4	6.9	11
	infants (3–12 mo)	5.7	3.5	1.2	0.76
	toddlers	9.6	5.9	2.1	1.3
	children	2.8	1.8	0.61	0.38
	teens	1.5	0.95	0.33	0.21
	adults	1.5	0.93	0.33	0.20
scenario B	infants (0–3 mo)	25	8.8	6.9	11
	infants (3–12 mo)	4.4	0.96	1.4	0.82
	toddlers	7.4	1.6	2.3	1.4
	children	2.2	0.48	0.69	0.41
	teens	1.2	0.26	0.38	0.22
	adults	1.2	0.25	0.37	0.22
scenario C	infants (0–3 mo)	24	8.7	6.8	11
	infants (3–12 mo)	2.7	0.59	0.78	0.60
	toddlers	4.6	0.99	1.3	1.0
	children	1.4	0.29	0.39	0.30
	teens	0.74	0.16	0.21	0.16
	adults	0.72	0.15	0.21	0.16
bottom-up	infants (0–3 mo)	44	13	6.7	21
	infants (3–12 mo)	2.8	1.7	0.32	0.26
	toddlers	1.2	0.80	0.15	0.12
	children	1.0	0.63	0.12	0.093
	teens	0.64	0.39	0.072	0.058
	adults	0.50	0.30	0.055	0.044

References

Abdallah MAE, Tilston E, Harrad S, Collins C. 2012. In vitro assessment of the bioaccessibility of brominated flame retardants in indoor dust using a colon extended model of the human gastrointestinal tract. J Environ Monitor 14:3276–3283.

ABS (Australian Bureau of Statistics). 1998. National nutrition survey: Nutrient intakes and physical measurements, Australia, 1995. Canberra:ABS. Available: http://www.abs.gov.au/AUSSTATS/abs@.nsf/DetailsPage/4805.01995?OpenDocument [accessed 17 February 2015].

FSANZ (Food Standards Australia New Zealand). 2007. Polybrominated diphenyl ethers (PBDE) in food in Australia. Canberra:FSANZ. Available: http://www.foodstandards.gov.au/science/surveillance/Pages/fsanzstudyofbrominat4997.aspx [accessed 17 February 2015].

Horton A, Murray F, Bulsara M, Hinwood A, Farrar D. 2006. Personal monitoring of benzene in Perth, Western Australia: The contribution of sources to non-industrial personal exposure. Atmos Environ 40:2596–2606.

Lorber M. 2008. Exposure of Americans to polybrominated diphenyl ethers. J Expo Sci Environ Epidemiol 18:2–19.

Moser GA, McLachlan MS. 2001. The influence of dietary concentration on the absorption and excretion of persistent lipophilic organic pollutants in the human intestinal tract. Chemosphere 45:201–211.

Roper CS, Simpson AG, Madden S, Serex TL, Biesemeier JA. 2006. Absorption of (^{14}C)-tetrabromodiphenyl ether (TeBDE) through human and rat skin in vitro. Drug Chem Toxicol 29:289–301.

Toms L-ML, Bartkow ME, Symons R, Paepke O, Mueller JF. 2009a. Assessment of polybrominated diphenyl ethers (PBDEs) in samples collected from indoor environments in South East Queensland, Australia. Chemosphere 76:1730–178.

Toms L-ML, Guerra P, Eljarrat E, Barcelo D, Harden FA, Hobson P, et al. 2012. Brominated flame retardants in the Australian population: 1993–2009. Chemosphere 89:398–403.

Toms L-ML, Hearn L, Kennedy K, Harden F, Bartkow M, Temme C, et al. 2009b. Concentrations of polybrominated diphenyl ethers (PBDEs) in matched samples of human milk, dust and indoor air. Environ Int 35:864–869.

Trudel D, Scheringer M, von Goetz N, Hungerbühler K. 2011. Total consumer exposure to polybrominated diphenyl ethers in North America and Europe. Environ Sci Technol 45:2391–2397.

U.S. EPA (Environmental Protection Agency). 2011. Exposure Factors Handbook. EPA/600/R-10/030. Available: http://cfpub.epa.gov/ncea/risk/recordisplay.cfm?deid=236252 [accessed 17 February 2015].

Verner MA, Sonneborn D, Lancz K, Muckle G, Ayotte P, Dewailly E, et al. 2013. Toxicokinetic modeling of persistent organic pollutant levels in blood from birth to 45 months of age in longitudinal birth cohort studies. Environ Health Perspect 121:131–137.

WHO (World Health Organization). 2002. Nutrient adequacy of exclusive breastfeeding for the term infant during the first six months of life. Geneva:WHO. Available: http://www.who.int/maternal_child_adolescent/documents/9241562110/en/
[accessed 17 February 2015].

WHO (World Health Organization) Multicentre Growth Reference Study Group. 2006. WHO child growth standards: Length/height-for-age, weight-for-age, weight-for-length, weight-for-height and body mass indext-for-age: Methods and development. Geneva:WHO. Available: http://www.who.int/childgrowth/standards/technical_report/en/ [accessed 17 February 2015].
